# A Comparison of Short-term and Long-term Air Pollution Exposure Associations with Mortality in Two Cohorts in Scotland

**DOI:** 10.1289/ehp.1104509

**Published:** 2012-06-06

**Authors:** Iain J. Beverland, Geoffrey R. Cohen, Mathew R. Heal, Melanie Carder, Christina Yap, Chris Robertson, Carole L. Hart, Raymond M. Agius

**Affiliations:** 1 Department of Civil Engineering, University of Strathclyde, Glasgow, United Kingdom; 2 Edinburgh, United Kingdom; 3 School of Chemistry, University of Edinburgh, Edinburgh, United Kingdom; 4 Centre for Occupational and Environmental Health, University of Manchester, Manchester, United Kingdom; 5 Department of Mathematics and Statistics, University of Strathclyde, Glasgow, United Kingdom; 6 Health Protection Scotland, Glasgow, United Kingdom; 7 International Prevention Research Institute, Lyon, France; 8 Institute of Health and Wellbeing, Public Health, University of Glasgow, Glasgow, United Kingdom

**Keywords:** air, associations, cohort, exposure–mortality, long term, pollution, short term, time-series

## Abstract

Background: Air pollution–mortality risk estimates are generally larger at longer-term, compared with short-term, exposure time scales.

Objective: We compared associations between short-term exposure to black smoke (BS) and mortality with long-term exposure–mortality associations in cohort participants and with short-term exposure–mortality associations in the general population from which the cohorts were selected.

Methods: We assessed short-to-medium–term exposure–mortality associations in the Renfrew–Paisley and Collaborative cohorts (using nested case–control data sets), and compared them with long-term exposure–mortality associations (using a multilevel spatiotemporal exposure model and survival analyses) and short-to-medium–term exposure–mortality associations in the general population (using time-series analyses).

Results: For the Renfrew–Paisley cohort (15,331 participants), BS exposure–mortality associations were observed in nested case–control analyses that accounted for spatial variations in pollution exposure and individual-level risk factors. These cohort-based associations were consistently greater than associations estimated in time-series analyses using a single monitoring site to represent general population exposure {e.g., 1.8% [95% confidence interval (CI): 0.1, 3.4%] vs. 0.2% (95% CI: 0.0, 0.4%) increases in mortality associated with 10-μg/m^3^ increases in 3-day lag BS, respectively}. Exposure–mortality associations were of larger magnitude for longer exposure periods [e.g., 3.4% (95% CI: –0.7, 7.7%) and 0.9% (95% CI: 0.3, 1.5%) increases in all-cause mortality associated with 10-μg/m^3^ increases in 31-day BS in case–control and time-series analyses, respectively; and 10% (95% CI: 4, 17%) increase in all-cause mortality associated with a 10-μg/m^3^ increase in geometic mean BS for 1970–1979, in survival analysis].

Conclusions: After adjusting for individual-level exposure and potential confounders, short-term exposure–mortality associations in cohort participants were of greater magnitude than in comparable general population time-series study analyses. However, short-term exposure–mortality associations were substantially lower than equivalent long-term associations, which is consistent with the possibility of larger, more persistent cumulative effects from long-term exposures.

The magnitude of adverse health effects associated with air pollution has been the subject of extensive research (Brook et al. 2010; Pope and Dockery 2006). An aspect of epidemiological research of importance to policy makers is the time scale over which adverse effects are most apparent (Künzli 2005). Discrepancies between the magnitudes of short-to-medium–term exposure effects estimated at time scales of days to weeks, and of long-term exposure effects estimated by following cohorts over years to decades, has been a particular concern (Pope 2007).

In longitudinal cohort studies, long-term average pollution exposures of individual cohort participants are estimated, and the contribution of the geographical variation among these exposures to the risk of adverse events over the entire follow-up period is determined by survival analysis, adjusting for characteristics that may affect disease risk. In contrast, many studies of short-term exposure effects have examined relationships between daily counts of deaths in a given population and daily pollutant and meteorological variables recorded at central monitoring sites using Poisson regression modeling and generalizations thereof. This type of time-series study has limited capacity to take account of spatial variation in individual exposures or to investigate the possible modification of pollution effects by individual risk factors such as age, smoking history, and previous disease, although analysis using case-crossover methods (Carracedo-Martinez et al. 2010) may reduce some of these difficulties. The estimates of air pollution effects from long-term exposure studies involving cohorts have generally been considerably larger than those from short-term time-series studies even after adjusting for other individual risk factors (Pope 2007). It is plausible that larger magnitudes of association between long-term exposure and mortality may be attributable to cumulative effects that increase the sensitivity of highly exposed population subgroups.

However, there have been few studies comparing the estimated effects of the same pollutant on the same population at both short- and long-term time scales. In the present study, we present such a comparison, using mortality data from approximately 25 years of follow-up on two Scottish cohorts with a combined size of nearly 20,000 participants and estimates of temporal and spatial variations in black smoke (BS) air pollution in the contiguous urban area of Glasgow, Paisley, and Renfrew in central Scotland, United Kingdom (the Glasgow conurbation). Short-term effects were estimated using a nested case–control approach in which the pollution experienced by each case immediately before death was compared with that experienced by controls of the same age in approximately the same time period and at approximately the same time of year. The effects of long-term pollution exposure were estimated from a detailed spatiotemporal exposure model. Additionally, short-term exposure–mortality associations were estimated using conventional Poisson regression time-series models in the urban population from which the majority of cohort participants were selected.

## Methods

*Study population.* The Renfrew–Paisley cohort was recruited from residents 45–64 years of age from Renfrew and Paisley in west central Scotland, which are contiguous to each other and to the city of Glasgow. The cohort comprised 78% of the target population, with 15,402 participants screened between 1972 and 1976 (Hawthorne et al. 1995). The Collaborative cohort is an occupational cohort of 7,028 participants recruited from 27 workplaces in central Scotland between 1970 and 1973 (Davey Smith et al. 1998). These two cohorts were designed for study together, and in this context are referred to as the Midspan study (Hart et al. 2005). In the present study, we examined short- and medium-term exposure–mortality associations in 15,331 participants in the Renfrew–Paisley cohort with complete postal code information and 3,818 Collaborative cohort participants (35–64 years of age at recruitment) residing in the Glasgow urban area with complete postal code information. To provide an indication of geographical scale the contiguous conurbation of Glasgow, Paisley, and Renfrew can be encompassed within a radius of 12 km, with Renfrew and Paisley encompassed by radii of 1.5 and 3.5 km, respectively, within this 12-km radius. Participants in both cohorts underwent physical examinations at recruitment and completed similar detailed questionnaires. Baseline variables used in our analyses included marital status (married, single, widowed, or other), smoking status (never; ex-smoker; current smoker of 1–14, 15–24, or > 25 cigarettes/day; or pipe/cigar smoker), occupational social class [categorized as I, II, III non-manual, III manual, IV, or V according to the Registrar General’s Classification (General Register Office 1966)], body mass index, systolic blood pressure, and total plasma cholesterol.

Follow-up of mortality using linkage to the National Health Service Central Register was available up to April 1998 for both cohorts. In addition to deaths from any cause, mortality from cardiovascular causes (ICD-9 codes 410–414, 426–429, 434–440) and respiratory causes (ICD-9 codes 480–487, 490–496) were considered [coded to the *International Classification of Diseases, Revision 9* (ICD-9; World Health Organization 1977).

*Pollution exposure.* Analysis of short-term pollution exposure was based on records of daily BS concentration between 1974 and 1998 at a single monitoring site close to the center of the Glasgow conurbation, which had few missing values and was situated in a residential area with medium- to high-density housing interspersed with some industrial undertakings [UK classification A2/B2 (Department for Environment Food and Rural Affairs 2005)]. From a review of approximately 10 potential monitoring sites in the conurbation, taking into account prevailing winds, population distribution, site classification and data capture during 1974–1998, this was considered the most appropriate site to use to estimate temporal variations in background air pollution. Three averaging periods were used for assessing short- and medium-term exposure: a 3-day average over the 3 days preceding the day of death; a 7-day average over the day of death and the preceding 6 days; and a 31-day average over the day of death and the preceding 30 days.

Analysis of long-term pollution exposure for the decade 1970–1979 was based on records from 181 monitoring sites distributed widely over central Scotland. Many of these sites had long periods of missing data. After 1979, the number of sites was greatly reduced.

The use of the BS metric (derived from the darkness of particulate matter collected on a filter through which air has been sampled) is well established in historical UK monitoring networks and is considered to be a good marker for traffic and other combustion-related urban air pollution through similar measurements of filter reflectance (Hochadel et al. 2006; Janssen et al. 2011).

*Temperature data.* We obtained daily averages of hourly temperatures from 0700–2300 hours at the UK meteorological office site at Glasgow airport, and calculated 3-, 7-, and 31-day temperature averages as for BS. We modeled temperature effects using a bilinear model which assumed a “knot” at 11°C with differential linear effects of temperature change below and above the knot, based on a previous analysis of temperature effects on mortality in the general population of Glasgow (Carder et al. 2005). We did not adjust for spatial variation in temperature when estimating long-term effects because we assumed that relatively shallow spatial gradients in long-term average temperature would not confound air pollution–mortality associations.

*Models for short-term pollution effects using centrally estimated exposures.* Because there was only one monitoring site with adequate data for the whole study period, a conventional survival analysis model could not be used to study short-term effects as all cohort participants would have been assigned the same values for short-term pollution exposure at a given time.

We therefore constructed nested case–control data sets as follows. For each death in a cohort, we considered controls selected randomly from among the cohort participants who lived at least as long as the case. Each control had an associated date, namely the date when the control reached the exact age at which the case died. If the control date was outside the follow-up period of the cohort, that control could not be used. We restricted controls to persons of the same sex who lived longer than the case, whose control date was in the same calendar month as the case death, and whose date of birth was within 1 calendar year of the date of birth of the case. Up to 9 controls were randomly selected from the full set available for each case. For all-cause deaths, 96% (8,342/8,700) of cases in the Renfrew–Paisley cohort and 76% (1,160/1,524) of cases in the Collaborative cohort had the full complement of 9 controls and 99% and 92%, respectively, had at least 4 controls. Six nested case–control data sets of this type were created for three case outcomes (all-cause, cardiovascular, and respiratory mortality) in the two cohorts. We then used conditional logistic regression to compare the pollution experienced by the case immediately before death and that experienced by controls of the same age in approximately the same time period and at approximately the same time of year, with adjustment for potential confounding variables measured at baseline: smoking history (six levels), social class (six levels), body mass index (five quintiles), marital status (four levels), systolic blood pressure (linear), and total cholesterol (linear). This conditional logistic regression model does not correspond to a survival model because generally different controls are in each case–control set. However, because of the correspondence between the likelihoods for proportional hazards and conditional logistic regression modeling (Collett 1994), the BS effect parameter, representing the ratio between the odds of being a case versus a control for a given increment in exposure, may be interpreted as an approximate estimate of the hazard ratio in a proportional hazards survival model where the level of BS exposure is age dependent.

*Models for long-term pollution effects.* Long-term exposures to BS were estimated for 1970–1979 using a multilevel spatiotemporal model that used a combination of time-series and spatial smoothing techniques to model monthly BS at 181 monitoring sites (across the central part of Scotland including the Glasgow conurbation) simultaneously taking into account seasonal effects and local air quality predictors including altitude (*A*), household density within a 250-m radius (*HD*), distance to nearest major road (*MR*), and distance to an urban boundary (*UB*) (Beverland et al. 2012):


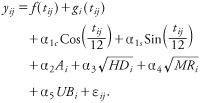


Here *y_ij_* denotes monthly mean ln(BS + 0.5) at site *i* at time *t_ij_* (where *i =* 1*…s* indexes sites, and *j* = 1…*n_i_* indexes observations within a site); *f*(*t_ij_*) is the BS temporal trend averaged over the population of all sites; *g_i_*(*t_ij_*) is the deviation of the *i*th site from the population mean at time *t_ij_* [*f*(*t_ij_*) and *g_i_*(*t_ij_*) were estimated using penalized linear splines]; sine and cosine terms model seasonal effects with α_1c_, α_1s_ as fixed-effect parameters; and α_2_*…*α_5_ are local air quality predictor fixed-effect parameters. The “within-site” error term, ε*_ij_*, representing the deviation of predicted mean ln(BS) from observed ln(BS), is assumed to be normally distributed. The multilevel model allowed estimation of coefficients between BS and local air quality predictors in the presence of missing data and hence was not dependent on imputation techniques (Beverland et al. 2012). The model allowed imputation of missing data at monitoring sites and subsequent estimation of long-term average BS pollution exposure for individual cohort participants based on geographical coordinates and the smoothed residual effects of estimated random intercepts and temporal trends at monitoring sites around each address location (with estimated individual exposures for 1970–1979 ranging from 6.4 to 55.3 μg/m^3^) (Yap et al. 2012).

We evaluated the multilevel model in a cross-validation study (Beverland et al. 2012). First we identified BS monitoring sites with > 80% data (39 sites) and imputed missing data with a site-specific time-series model with a flexible trend and month and day effects to give 39 sites with “complete” data. Ten-year geometric mean BS concentrations at these sites ranged from 8.9 to 48.2 μg/m^3^. We then created a “test data set” from 19 of these sites selected at random and a “training data set” from the remaining 20 sites together with the 142 sites with < 80% data coverage. The model was fitted to the training data set and then used to predict BS in the test data set. This cross-validation procedure was repeated 10 times with different random selections from the 39 complete data sites forming the test set. The average difference (± SE) between predicted and observed concentrations for test sites was 1.2 ± 1.10 μg/m^3^ and the root mean square difference was 6.8 μg/m^3^.

We estimated the effects of long-term BS exposure on mortality up to 1998 using Cox proportional hazards models, with baseline hazard functions stratified by 1-year age groups and sex while incorporating the same individual baseline covariates as in the short-term effects models described above (Yap et al. 2012).

*Models for short-term pollution effects using individually weighted exposures.* We calculated the ratios of individual participants’ geometric mean long-term exposures to BS (multilevel model estimates for 1970–1979 at address postal codes) to the 1970–1979 geometric mean BS at the central monitoring site (27.9 μg/m^3^). We applied these individual multiplying factors to the series of daily values at the central site to estimate individual-specific time-series over the period 1974–1998 reflecting both spatial and temporal variation. From these series, we calculated individual daily values of the 3-, 7-, and 31-day averages of BS exposure for the conditional logistic regressions applied to the same nested case–control data sets as described above.

*Time-series modeling.* BS effects were also estimated using Poisson regression analysis of time-series of daily mortality at ≥ 50 years of age (to provide an approximate match to the aging cohort during this period) from 1974 to 1998 in the general population (approximately 1 million persons) of the contiguous Glasgow plus Renfrew–Paisley conurbation. Briefly, the model incorporated smooth functions of time (natural cubic splines, with seven degrees of freedom per year, to capture seasonal and other long-term effects), indicator variables for day of the week, and measures of BS (at single central monitoring site) and temperature at lags ≤ 30 days grouped into 6-day periods (Carder et al. 2008). A simple Poisson model was assumed because there was no evidence of overdispersion.

## Results

Table 1 shows the numbers of cases with known exposure data available by decade, and compares mean values of 3-day mean BS over cases and controls in each of the analysis data sets. In the Renfrew–Paisley area, the mean exposure for cases exceeded that for controls in all comparisons except for all-cause deaths in the 1970s. This suggests, *prima facie*, that there was an association between short-term BS exposure and mortality. We observed no clear trend between decades, nor systematic differences by cause of death. There was an overall positive difference between case and control exposure for each cause of death for the Collaborative cohort, but the differences by decade were quite variable.

**Table 1:** Mean BS concentrations (μg/m^3^) over preceding 3 days for cases and controls.^a^

Cohort/mortality/year of case death		nb	Cases	Controlsc	Cases – controls
Renfrew–Paisley									
All-cause									
	1971–1979		1,046		37.36		37.86		–0.50
	1980–1989		2,823		19.90		18.63		1.27
	1990–1998		4,186		11.38		11.02		0.36
	All		8,055		17.74		17.17		0.57
Cardiovascular									
	1971–1979		446		38.59		38.08		0.51
	1980–1989		1,347		19.25		18.21		1.04
	1990–1998		1,850		11.64		11.23		0.41
	All		3,643		17.75		17.10		0.65
Respiratory									
	1971–1979		68		44.51		43.38		1.13
	1980–1989		187		22.74		20.35		2.39
	1990–1998		396		11.05		10.60		0.45
	All		651		17.90		16.83		1.07
Collaborative									
All-cause									
	1971–1979		242		42.51		38.06		4.45
	1980–1989		603		18.98		19.05		–0.07
	1990–1998		571		12.65		12.59		0.06
	All		1,416		20.45		19.70		0.75
Cardiovascular									
	1971–1979		120		43.22		40.63		2.59
	1980–1989		278		19.11		18.52		0.59
	1990–1998		226		11.52		12.77		–1.25
	All		624		21.00		20.69		0.31
Respiratory									
	1971–1979		7		41.43		48.07		–6.64
	1980–1989		50		22.75		21.43		1.32
	1990–1998		54		12.87		13.09		–0.22
	All		111		19.12		19.05		0.07
aEach case–control set was given equal weight (i.e., the mean control exposure was calculated in each set and then these means were averaged). bNumber of deaths (cases); about 7% of deaths were excluded because the 3-day mean of BS was missing. cControls of same sex born within 1 year of case and reaching control date in the same calendar month as case death.									

Table 2 presents the estimated short-term effects for each cohort and mortality outcome, using three averaging periods of exposure, and either using or not using the individual spatial weights. In each case, the pollution effect (percent increase in odds of mortality for a 10-μg/m^3^ increment in average BS concentration) was estimated with and without adjusting for the risk factors. The table illustrates how the estimates changed with increasing degrees of adjustment for risk factors, spatial weighting, and bilinear temperature.

**Table 2:** Estimated percent change^a^ in mortality with a 10-μg/m^3^ increase in BS based on nested case–control analyses according to exposure time period (BS averaging period), cohort, and model.

Days –1 to –3			Days 0 to –6			Days 0 to –30		
Percent change (95% CI)	p-Value	Percent change (95% CI)	p-Value	Percent change (95% CI)	p-Value
All-cause													
Renfrew–Paisley													
	CSBS		1.4 (0.3, 2.6)		0.011		1.7 (0.2, 3.2)		0.028		4.1 (1.2, 7.0)		0.005
	CSBS + risk factors		1.5 (0.4, 2.6)		0.009		1.7 (0.2, 3.3)		0.027		3.7 (0.9, 6.7)		0.011
	CSBS + risk factors + temperature		1.0 (–0.2, 2.3)		0.100		1.1 (–0.7, 2.8)		0.231		1.1 (–2.3, 4.7)		0.516
	IWBS		2.3 (0.7, 3.8)		0.003		2.9 (0.9, 5.0)		0.005		6.3 (2.7, 10.0)		0.001
	IWBS + risk factors		2.3 (0.8, 3.9)		0.003		3.0 (0.9, 5.1)		0.004		5.8 (2.2, 9.6)		0.001
	IWBS + risk factors + temperature		1.8 (0.1, 3.5)		0.040		2.3 (0.0, 4.6)		0.051		3.4 (–0.7, 7.7)		0.106
Collaborative													
	CSBS		1.3 (–1.0, 3.7)		0.270		1.7 (–1.6, 5.1)		0.305		–2.0 (–8.0, 4.4)		0.531
	CSBS + risk factors		0.6 (–1.9, 3.2)		0.636		0.5 (–2.9, 4.1)		0.771		–3.1 (–9.2, 3.4)		0.345
	CSBS + risk factors + temperature		–0.1 (–2.9, 2.7)		0.921		–0.7 (–4.5, 3.3)		0.736		–5.0 (–12, 2.6)		0.194
	IWBS		2.6 (0.3, 4.8)		0.024		3.4 (0.4, 6.5)		0.027		3.8 (–1.1, 8.9)		0.128
	IWBS + risk factors		1.6 (–0.8, 4.1)		0.188		1.8 (–1.4, 5.1)		0.276		2.0 (–3.0, 7.2)		0.446
	IWBS + risk factors + temperature		1.1 (–1.4, 3.8)		0.390		1.1 (–2.3, 4.7)		0.528		2.0 (–3.4, 7.6)		0.482
Cardiovascular													
Renfrew–Paisley													
	CSBS		1.8 (0.1, 3.5)		0.042		2.9 (0.6, 5.3)		0.013		7.4 (3.1, 11.9)		0.001
	CSBS + risk factors		1.4 (–0.3, 3.1)		0.109		2.6 (0.2, 5.0)		0.031		6.5 (2.1, 11.1)		0.003
	CSBS + risk factors + temperature		0.9 (–1.0, 2.8)		0.351		1.5 (–1.1, 4.2)		0.271		2.9 (–2.3, 8.4)		0.276
	IWBS		2.4 (0.1, 4.7)		0.043		3.8 (0.7, 7.0)		0.016		8.7 (3.2, 14.5)		0.002
	IWBS + risk factors		2.0 (–0.3, 4.4)		0.089		3.5 (0.4, 6.8)		0.027		8.2 (2.6, 14.1)		0.004
	IWBS + risk factors + temperature		1.4 (–1.2, 4.0)		0.287		2.2 (–1.3, 5.8)		0.225		4.1 (–2.2, 10.7)		0.208
Collaborative													
	CSBS		0.3 (–3.2, 3.9)		0.875		1.7 (–3.2, 6.9)		0.507		–3.5 (–12, 6.0)		0.454
	CSBS + risk factors		–1.3 (–4.8, 2.4)		0.484		–0.3 (–5.1, 4.9)		0.919		–3.9 (–13, 6.0)		0.424
	CSBS + risk factors + temperature		–2.1 (–6.0, 1.9)		0.303		–1.1 (–6.5, 4.7)		0.712		–5.3 (–16, 6.1)		0.349
	IWBS		1.5 (–1.9, 4.9)		0.394		3.0 (–1.6, 7.8)		0.202		1.3 (–5.8, 8.8)		0.735
	IWBS + risk factors		–0.2 (–3.6, 3.4)		0.930		1.0 (–3.6, 5.8)		0.681		0.3 (–7.0, 8.1)		0.944
	IWBS + risk factors + temperature		–0.6 (–4.3, 3.2)		0.747		0.6 (–4.4, 5.9)		0.820		0.4 (–7.5, 8.9)		0.930
Respiratory													
Renfrew–Paisley													
	CSBS		2.8 (–1.3, 7.1)		0.181		3.1 (–2.3, 8.9)		0.270		10.1 (–0.6, 21.9)		0.065
	CSBS + risk factors		1.7 (–2.6, 6.1)		0.449		1.9 (–3.6, 7.7)		0.503		5.6 (–4.8, 17.2)		0.304
	CSBS + risk factors + temperature		–0.2 (–5.0, 4.7)		0.920		–0.6 (–6.7, 6.0)		0.857		–0.6 (–12, 12.9)		0.932
	IWBS		3.8 (–1.5, 9.4)		0.167		5.1 (–2.0, 12.7)		0.167		19.4 (5.2, 35.5)		0.006
	IWBS + risk factors		2.0 (–3.6, 7.8)		0.495		3.0 (–4.2, 10.6)		0.425		12.1 (–1.4, 27.4)		0.082
	IWBS + risk factors + temperature		–0.4 (–6.4, 6.1)		0.912		0.2 (–7.6, 8.6)		0.964		7.2 (–7.5, 24.2)		0.358
Collaborative													
	CSBS		0.0 (–9.6, 10.7)		0.998		0.7 (–12, 15.0)		0.922		–24 (–44, 3.2)		0.079
	CSBS + risk factors		0.9 (–8.9, 11.6)		0.869		0.7 (–12, 14.9)		0.923		–23 (–43, 4.1)		0.089
	CSBS + risk factors + temperature		–1.2 (–12, 10.4)		0.826		–2.8 (–16, 12.8)		0.704		–29 (–50, 0.8)		0.055
	IWBS		2.6 (–5.8, 11.8)		0.556		4.1 (–6.6, 16.1)		0.463		–16 (–34, 6.5)		0.151
	IWBS + risk factors		2.4 (–6.1, 11.7)		0.589		2.5 (–8.2, 14.5)		0.656		–17 (–34, 4.6)		0.114
	IWBS + risk factors + temperature		1.1 (–7.8, 10.9)		0.817		0.7 (–11, 13.4)		0.914		–19 (–38, 4.0)		0.097
Abbreviations: CI, confidence interval; CSBS, BS at (single) central monitoring site; IWBS, individually weighted BS from ratio of individual participant's long-term geometric mean exposure to BS, estimated by multilevel model, to geometric mean BS at the central monitoring site (time-series of daily values at central site were multiplied by individual ratios to produce individual-specific time-series for 1974–1998 reflecting both spatial and temporal variation); risk factors, adjustment for potential confounding variables measured at baseline—smoking history, social class, body mass index, marital status, systolic blood pressure, and total cholesterol; temperature, linear effects of temperature > and < 11°C. aEstimates in this table are odds ratios (ORs) from conditional logistic regression models comparing the pollution experienced by the case immediately before death and that experienced by controls of the same age in approximately the same time period and at approximately the same time of year.													

*Estimated effects on all-cause mortality.* Generally positive associations between short-term BS exposure and all-cause mortality were estimated for both cohorts. Significant effects were found in the Renfrew–Paisley cohort, particularly when the individually weighted exposures were used. Adjusting for risk factors and temperature, the effect of 3-day mean BS was estimated as a 1.0% [95% confidence interval (CI): –0.2, 2.3%] increase in hazard using centrally estimated exposures and a 1.8% (95% CI: 0.1, 3.5%) increase using individually weighted exposures (Table 2). Corresponding estimates of the effect of a 31-day mean BS were higher: 1.1% (95% CI: –2.3, 4.7%) and 3.4% (95% CI: –0.7, 7.7%) for centrally estimated and individually weighted exposures respectively. Exposure–mortality associations were generally smaller and nonsignificant in the Collaborative cohort, with some nonsignificant negative associations for central site exposure over the 31-day period. Apart from the smaller number of Collaborative cohort participants included in our analyses, possible reasons for the different results in the two cohorts are that they differed with regard to age, social class, employment, geographical distribution, and election.

Adjusting for risk factors did not greatly change effect estimates of 3- and 7-day mean BS in the Renfrew–Paisley cohort, but reduced those of the 31-day mean. Adjusting for temperature made a substantial difference, reducing estimates by about 33% for the 3- and 7-day means and considerably more for the 31-day mean at the central site.

*Estimated effects on cardiovascular mortality.* The pattern of associations with cardiovascular mortality was similar to that for all-cause mortality, with higher estimates for the longer exposure periods and when individually weighted exposures were used. For the Renfrew–Paisley cohort the 31-day BS mean was associated with larger mortality increases than the 3- and 7-day means [after adjusting for risk factors, temperature and spatial variation, a 10-μg/m^3^ increment in average BS exposure over the previous 31 days was associated with a 4.1% (95% CI: –2.2, 10.7%) increase in cardiovascular mortality for this cohort]. In the Collaborative cohort there were no statistically significant associations and the estimated effect sizes were small and, in some instances, negative.

*Estimated effects on respiratory mortality.* There were very few statistically significant associations and estimates generally had very wide confidence intervals, making any inferences extremely tentative. In the Collaborative cohort, the 31-day exposures were associated with strongly negative, although nonsignificant, effects; whereas in the Renfrew–Paisley cohort, the 31-day exposures were associated with large positive, but generally nonsignificant, effects. For 3- and 7-day mean BS, the estimates were generally positive before adjusting for temperature.

*Comparison with long-term effect estimates.* Table 3 compares selected estimates of the short and medium-term effects of BS on mortality from Table 2 with estimated long-term effects in the same cohorts, obtained from proportional hazards modeling (Yap et al. 2012). In the Renfrew–Paisley cohort long-term pollution exposure–mortality associations were substantially greater in magnitude than short-term exposure–mortality associations for all three categories of mortality. Long-term effects were much smaller in the Collaborative cohort than in the Renfrew–Paisley cohort and were nonsignificant.

**Table 3:** Comparison of estimated magnitudes of associations [percent change (95% CI)] between short- and long-term exposure to BS and mortality in the Renfrew–Paisley and Collaborative cohorts and in the population > 50 years of age of Glasgow, Renfrew, and Paisley conurbation with follow-up to 1998.

Mortality/population group	Short-term (3-day)a,b	Medium-term (31-day)a,b	Long-term (1970–1979)c
All-cause						
Time-seriesa		0.2 (0.0, 0.4)		0.9 (0.3, 1.5)		—
Renfrew–Paisley cohortb		1.8 (0.1, 3.5)		3.4 (–0.7, 7.7)		10 (4, 17)
Collaborative cohortb,d		1.1 (–1.4, 3.8)		2.0 (–3.4, 7.6)		1 (–4, 6)
Combined cohorte		1.6 (0.2, 3.0)		2.9 (–0.5, 6.2)		5 (1, 9)
Cardiovascular						
Time-seriesa		0.1 (–0.2, 0.4)		0.3 (–0.7, 1.2)		—
Renfrew–Paisley cohortb		1.4 (–1.2, 4.0)		4.1 (–2.2, 10.7)		11 (1, 22)
Collaborative cohortb,d		–0.6 (–4.3, 3.2)		0.4 (–7.5, 8.9)		3 (–5, 12)
Combined cohorte		0.8 (–1.4, 2.9)		2.7 (–2.4, 7.8)		7 (0, 13)
Respiratory						
Time-seriesa		0.3 (–0.2, 0.8)		3.1 (1.4, 4.9)		—
Renfrew–Paisley cohortb		–0.4 (–6.4, 6.1)		7.2 (–7.5, 24.2)		26 (2, 55)
Collaborative cohortb,d		1.1 (–7.8, 10.9)		–19.5 (–37.7, 4.0)		–3 (–21, 18)
Combined cohorte		0.1 (–5.1, 5.3)		–2.6 (–15.2, 10.0)		11 (–3, 28)
Table details percent increases in mortality associated with 10-μg/m3 increments in average BS. aRate ratios estimated by Poisson regression modeling (adjusted for temperature) for population > 50 years of age in the contiguous Glasgow, Renfrew, and Paisley conurbation (Beverland et al. 2007). bOdds ratios estimated by conditional logistic regression modeling (adjusted for temperature) on matched case–control sets and adjusted for baseline risk factors (smoking history, social class, body mass index, marital status, systolic blood pressure, and total cholesterol) and spatial variation (Table 2). cHazard ratios estimated by Cox regression modeling using long-term exposures estimated from the spatiotemporal model and adjusted for baseline risk factors listed in footnote b. dShort-term effects were estimated for the Glasgow conurbation subset of the Collaborative cohort (n = 3,818); long-term effects were estimated for the full Collaborative cohort (n = 6,680). eCombined cohort risk estimates computed from individual cohort estimates (as outlined in above footnotes) using weights proportional to the inverse variance of risk estimates in individual cohorts.						

Table 3 also displays estimates of short-term associations derived from time-series modeling of the daily variation in mortality for the entire population of the Glasgow conurbation that was > 50 years of age (Beverland et al. 2007). The time-series estimates were smaller than the corresponding short-term estimates in the two cohorts, but they had narrower confidence intervals, being based on a much larger population.

## Discussion

There have been a limited number of studies comparing short- and long-term effects of air pollution in related study populations [e.g., Klemm and Mason (2003), Laden et al. (2006), and Schwartz et al. (1996) as reviewed by Pope (2007)]. However, to the best of our knowledge, direct estimation of the short-term effects of air pollution within a cohort study has rarely been attempted. In our case–control analysis, pollution on the days preceding the death of a cohort participant was compared with the pollution preceding the days when the controls reached the age at which the case died. We matched for sex, month of death, and within 1 year of birth and adjusted for several individual-level risk factors strongly associated with mortality. We also adjusted exposure estimates for spatial variation in personal exposure, and adjusted for temperature. This methodology might be expected to produce estimates of short-term effects that are more realistic than those available from time-series modeling and more comparable with the estimates available from survival analysis of long-term follow-up in cohorts. We compared the estimated effects of short- and medium-term exposure on mortality within cohort participants directly to estimated effects of long-term exposure on mortality within the same cohorts (Yap et al. 2012). We also compared the estimated effects of short- and medium-term exposure with rate–ratio estimates obtained from Poisson regression analysis of daily mortality within the general population from which the majority of cohort participants were selected (Beverland et al. 2007).

For the Renfrew–Paisley cohort, larger exposure–mortality associations were noted in the nested case–control analyses than in the time-series analyses using a single monitoring site (Table 3); the difference being consistent for both all-cause and cardiovascular mortality. This may be a result of more realistic exposure classification in the cohort-based analyses (Armstrong 1998; Sheppard et al. 2012). In both nested case–control and time-series analyses, the pollution–mortality associations were larger for medium-term (31-day) compared to short-term (3-day) averaging/lag periods.

However, the short- and medium-term effect estimates from the nested case–control analysis of the Renfrew–Paisley cohort were substantially smaller than the equivalent effects estimated for long-term exposure (Beverland et al. 2007; Yap et al. 2012), which is consistent with a review of exposure–outcome associations at different time scales (Pope 2007). For example, Yap et al. (2012) observed that a 10-μg/m^3^ increase in average BS over the decade 1970–1979 was associated with a 10% (95% CI: 4, 17%) increase in the hazard of all-cause mortality as compared with our estimate of a 1.8% (95% CI: 0.1, 3.4%) increase in hazard for a 10-μg/m^3^ increase in average BS over 3 days. Estimated associations for the Collaborative cohort were generally positive but much less consistent in magnitude, with no significant pollution effects observed in separate analyses of this cohort.

Our analyses of the effects of long-term and individually weighted short-term BS exposure assumed that the pattern of intraurban spatial variations in long-term average BS exposure in 1970–1979 was largely sustained over the subsequent 1980–1998 period, which could not be verified because of the substantial reduction in the BS monitoring network during this later period. Similar assumptions about relative invariance of spatial contrasts in long-term air pollution exposure have been made, by necessity, in almost all epidemiological studies of long-term intraurban air pollution exposure effects. These assumptions have been partly supported by observations of “stability” in spatial contrasts among measurement sites in a small number of studies (Eeftens et al. 2011; Hoek et al. 2008). An additional limitation is that exposure misclassification may have resulted from a lack of information about participant mobility.

Samoli et al. (2001) used time-series methods to estimate an average effect on all-cause mortality of 3.1% (95% CI: 2.4, 3.9%) for a 50-μg/m^3^ increase in BS averaged over the day of death and the previous day in four western European cities. A study using case–crossover analysis (Zeka et al. 2005), which has similarities to our approach, was applied to PM_10_ data for 20 U.S. cities between 1989 and 2000 and found that a 10-μg/m^3^ increase in PM_10_ averaged over the day of death and the previous 2 days was associated with 0.45% (95% CI: 0.25, 0.65%), 0.50% (95% CI: 0.25, 0.75%) and 0.87% (95% CI: 0.38, 1.36%) increases in all-cause, cardiovascular, and respiratory mortality, respectively. Using data from Dublin for 1980–1996, Goodman et al. (2004) estimated the effects of a 10-μg/m^3^ increase in 3-day average BS as 0.4% (95% CI: 0.3, 0.6%), 0.4% (95% CI: 0.2, 0.7%), and 0.9% (95% CI: 0.5, 1.2%) on all-cause, cardiovascular, and respiratory mortality, respectively. Although none of these studies used adjustments for spatial variation that were comparable with our approach, and they involved slightly different lag periods, the order of magnitude of the short-term effects is not inconsistent with ours, especially given the relatively wide confidence intervals associated with our estimates. However, while the use of spatially weighted exposures increased our effect estimates, even when using centrally measured exposures our case–control estimates for 3-day BS were generally higher than those observed in general population time-series and case–crossover analyses.

Another European multicity study using time-series methods reported city-specific all-cause mortality effects of a 10-μg/m^3^ increase in PM_10_ over a 40-day period ranging from –0.9% to 4.0% (Zanobetti et al. 2002). In our case–control analysis, we found associations of all-cause mortality with 10-μg/m^3^ increases in average BS over the day of death and the preceding 30 days of 3.4% (95% CI: –0.7, 7.7%) (Renfrew–Paisley after full adjustments) and 2.0% (95% CI: –3.4, 7.6%) (Collaborative), which are within this range and slightly greater than the equivalent effect of 1.1% (95% CI: 0.8, 1.3%) noted over a 40-day period in time-series analyses of BS and all-cause mortality in Dublin (Goodman et al. 2004).

The multicity studies cited above emphasize marked unexplained heterogeneity between city-specific estimates, so it is conceivable that there are specific conditions in the Glasgow conurbation that might explain differences between our results and those of others. It is also possible that BS gives a better measure than PM_10_ for the more damaging elements of air pollution, e.g., fine or ultrafine particles or specific transition metals (Heal et al. 2005; Hochadel et al. 2006; Janssen et al. 2011).

## Conclusions

After adjusting for individual-level risk factors, temperature, and geographical variation in BS pollution, short and medium-term BS exposure–mortality associations estimated using cohort-based nested case–control analyses were of greater magnitude than associations estimated for similar geographical areas using single-pollution-site time-series analyses. However, short and medium-term exposure–mortality associations were of substantially lower magnitude than long-term exposure-mortality associations observed in the same cohorts using survival analysis. These observations indicate the importance of intraurban variations in long-term pollution climates when estimating associations between exposure and mortality and suggest that public health impacts of air pollution may be dominated by long-term exposure determined by geographical differences in pollution climates.
